# Water Properties Influencing the Abundance and Diversity of Denitrifiers on *Eichhornia crassipes* Roots: A Comparative Study from Different Effluents around Dianchi Lake, China

**DOI:** 10.1155/2015/142197

**Published:** 2015-10-01

**Authors:** Neng Yi, Yan Gao, Zhenhua Zhang, Hongbo Shao, Shaohua Yan

**Affiliations:** Institute of Agricultural Resources and Environment and Institute of Agro-biotechnology, Jiangsu Academy of Agricultural Sciences, Nanjing 210014, China

## Abstract

To evaluate effects of environmental conditions on the abundance and communities of three denitrifying genes coding for nitrite (*nirK, nirS*) reductase and nitrous oxide (*nosZ*) reductase on the roots of *Eichhornia crassipes* from 11 rivers flowing into the northern part of Dianchi Lake. The results showed that the abundance and community composition of denitrifying genes on *E. crassipes* root varied with different rivers. The *nirK* gene copies abundance was always greater than that of *nirS* gene on the roots of *E. crassipes*, suggesting that the surface of *E. crassipes* roots growth in Dianchi Lake was more suitable for the growth of *nirK*-type denitrifying bacteria. The DGGE results showed significant differences in diversity of denitrifying genes on the roots of *E. crassipes* among the 11 rivers. Using redundancy analysis (RDA), the correlations of denitrifying microbial community compositions with environmental factors revealed that water temperature (*T*), dissolved oxygen (DO), and pH were relatively important environmental factors to modifying the community structure of the denitrifying genes attached to the root of *E. crassipes*. The results indicated that the specific environmental conditions related to different source of rivers would have a stronger impact on the development of denitrifier communities on *E. crassipes* roots.

## 1. Introduction

Phytoremediation technology using floating macrophytes (*Eichhornia crassipes*) performed very well in remediation eutrophic water body since* E. crassipes* is capable of assimilating large amount of nutrients efficiently [1–3]. During 2010–2012, large-scale confined growth of* E. crassipes* was used to remove pollutants (mainly N and P) from Dianchi Lake as well as the rivers connecting to the lake. Dianchi Lake is the sixth largest freshwater lake in China. There are more than 31 rivers, which carried wastewater discharged from different types of sewage treatment plants (STPs), agriculture, and domestic source, flowing into the lake. The macrophytes significantly improved water quality in both inflow rivers and Dianchi Lake [4].

To evaluate the contributions of water hyacinth to the removal of nitrogen from the lake, both assimilation and stimulated denitrification by the macrophyte are important since N-15 tracing experiment in labs indicated that the values of N-15 at.% excess of N_2_-N production were significantly (*p* < 0.05) higher with the growth of* E. crassipes* than that without [5, 6]. The presence of* E. crassipes* roots has positive effect on stimulating the activity, abundance, and diversity of denitrifiers. Studies have reported that plant rhizosphere enhanced bacterial abundance, activity, and diversity [7]. Previous studies also suggested that the root system of floating macrophyte could support the attachment of microorganism and enhance the growth and activity of bacteria for removing organic matter and nutrients [8].

Environmental conditions are also critically important in mediating the activity, abundance, and diversity of bacteria [9, 10]. The abundance and diversity of denitrifiers on* E. crassipes* roots grown in the rivers receiving wastewater with different water properties may vary with the variation of environmental factors. The abundance of the functional genes and community compositions of denitrifiers can be affected by many factors, such as water temperature, pH, DO, and nutrient concentrations. In the rivers with different sources of condensed pollutants and diverse physiochemical properties, the abundance and diversity of denitrifiers on the* E. crassipes* roots may be modified in various patterns.

Hence, in the present study, we investigated the abundance and diversity of denitrifying bacteria on* E. crassipes* roots in 11 rivers with different pollution sources in the north side of Dianchi Lake. It put an emphasis on understanding the interactions between the changes of environmental factors and the abundance and diversity of denitrifying bacteria attached to* E. crassipes* roots. It was expected that the results would shed some insight on how environmental factors and cultivation of* E. crassipes* mediate denitrification process in different eutrophic rivers flowing into the Dianchi Lake.

## 2. Materials and Methods

### 2.1. Site Description, Sampling, and Water Properties

**Figure 1:**
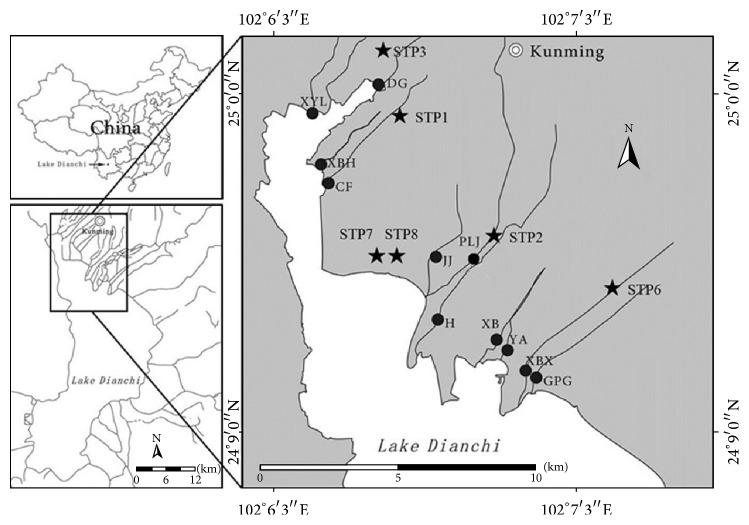
Sampling sites in rivers of Dianchi Lake. Black dot (●) is sampling site, and Pentagram (★) is sewage treatment plants (STP).

A total of 11 rivers around Dianchi Lake located from 24°9′ to 25°0′ latitude and 102°6′ to 102°7′ longitude were investigated (Figure 1). As shown in Figure 1, Xinbaoxiang (XBX), Daguan (DG), Chuanfang (CF), and Panlongjiang (PLJ) rivers receive effluents from sewage treatment plants (STPs). Haihe (H), Guangpugou (GPG), Jinjia (JJ), Xibahe (XBH), and Xinyunliang (XYL) rivers receive raw sewage from industrial, domestic, and agricultural sources. Xiaba (XB) and Yaoan (YA) rivers represent the same sewage origin from the same upstream (not sampled due to water hyacinth not being grown) but different tributaries separated at water treatment wetland named Wujia (not sampled due to water hyacinth not grown).

Water temperature, pH, and dissolved oxygen (DO) were measured* in situ* using portable meter (YSI ProPlus, USA). One-liter water samples were collected from each site with three replicates at 0–0.5 m of the water column of the eleven rivers using cylinder sampler in September 25, 2012. Total nitrogen (TN), ammonium (NH_4_^+^), nitrate (NO_3_^−^), nitrite (NO_2_^−^), and total soluble nitrogen (TSN) were analyzed using a SEAL AutoAnalyzer 3 (SEAL Analytical Co., Hampshire, UK). Mixed root samples were collected randomly with three replicates at each sampling site using sterile scissors and forceps and then stored in an ice box and taken back to the laboratory. Fresh roots (2 g) of* E. crassipes* were transferred into 200 mL sterile water. Bacteria attached to* E. crassipes* roots were detached by vigorous shaking for 30 min (18.3 Hz, Thermomixer Eppendorf) and filtered through a 0.45 *μ*m sterile filter. The resultant filtrates were filtered through 0.22 *μ*m Millipore membrane filters using a vacuum air pump and the membranes stored at −80°C for DNA extraction [5].

### 2.2. DNA Extraction

All the abovementioned membranes were cut into pieces with sterile scissors and used immediately for DNA extraction, which was performed using an E.Z.N.A. Water DNA Kit (OMEGA Bio-Tek Inc., Doraville, GA, USA) by following the manufacturer's instructions. The extracted DNA was stored in a −20°C freezer [5].

### 2.3. Real-Time Polymerase Chain Reaction (qPCR) Assay

Real-time quantitative PCR was performed to estimate the denitrifying bacteria abundance using the primers listed in Table 1. Real-time polymerase chain reaction (qPCR) was performed on ABI 7500 real-time System (Life Technologies, USA). Amplification was performed in triplicate in a total volume of 20 *μ*L reaction mixtures by using SYBR Premix Ex TaqTM (TIiRNaseH Plus) qPCR Kit as described by the suppliers (Takara Bio, Dalian, China). For each assay, three different PCR conditions were performed separately for the same sample by varying annealing temperature at either 54°C (*nirS- *Cd3Af/*nirS-*R3cd* and nosZ-*F/*nosZ-*1622R) or 58°C (*nirK-*F1aCu/*nirK-*R3Cu). The qPCR amplification was performed as follows: initial denaturation at 95°C for 2 min, followed by 35 cycles consisting of denaturation step at 95°C for 5 s, varying annealing temperature for 30 s, and elongation at 72°C for 30 s. The data were collected during the 72°C for 30 s step. Data was analyzed using the ABI 7500 software (Version 2.0.6, Life Technologies, USA). The parameter Ct (threshold cycle) was determined as the cycle number at which a statistically significant increase in the reporter fluorescence was detected. The standard curves for real-time PCR assays were developed as previously described [5].

### 2.4. PCR Amplification Denaturing Gradient Gel Electrophoresis (DGGE) Analysis

For denaturing gradient gel electrophoresis (DGGE) analysis, the PCR was performed in reaction mixtures including 1 *μ*L of template DNA, 5 *μ*L of 10 × PCR buffer, 1 *μ*L of dNTPs (10 mM each), 1 *μ*L of each primer (20 *μ*M) (Table 1), and 2 U of Taq polymerase (Takara Bio, Dalian, China) and adjusted to a final volume of 50 *μ*L with sterile deionized water. The reaction was performed in a Bio-Rad C1000 thermal cycler (Bio-Rad, USA) using different cycling conditions. The* nirK* gene (F1aCu/R3Cu-GC) PCR program was carried out with an initial denaturation at 94°C for 3 min, followed by 32 cycles of 94°C for 30 s, 58°C for 30 s, and 72°C for 45 s, followed by 72°C for 10 min, and ended at 10°C. The touchdown PCR amplification of* nirS* (Cd3Af/R3cd-GC) and* nosZ* (*nosZ*-F/*nosZ*1622R-GC) was performed as follows: 94°C for 2 min, followed by 10 cycles, 94°C for 30 s, and 57°C for 30 s in the initial cycle and at decreasing temperatures by 0.5°C/cycle until a temperature of 52°C was reached in the subsequent cycles. The extension step was performed at 72°C for 1 min. After the touchdown program, 30 cycles at 94°C for 30 s, 53°C for 30 s, and 72°C for 1 min, followed by 72°C for 10 min, and ended at 10°C.

The amplified products were pooled and resolved on DGGE gels using a Dcode system (Bio-Rad Laboratories, Hercules, USA). The purified PCR products (30 *μ*L) of* nirS*,* nirK*, and* nosZ* containing approximately equal amounts of PCR amplicons were loaded onto the 1 mm-thich 6% (w/v) polyacrylamide (37.5 : 1, acrylamide : bisacrylamide) gels with denaturing gradients of 50–75% for 15 h (*nirS*), 50–70% for 12 h (*nirK*), and 50–70% for 15 h (*nosZ*) (100% denaturant contains 7 mol/L urea and 40% (v/v) formamide). The gels were run in 1 × TAE (40 mM Tris-acetate and 1 mM EDTA) at 100 V and 60°C. The gel was silver-stained using protocol [11]. Polaroid pictures of the DGGE gels were scanned using an Epson Perfection V700 Photo scanner (Seiko Epson Corporation, Nagano, Japan). DGGE profiles were digitized after average background subtraction for the entire gel using Quantity One software (Version 4.5, Bio-Rad, USA) as previously described [5]. Digitized information from the DGGE banding profiles was used to calculate the diversity indices such as richness (*S*), which was determined from the number of bands in each lane, and Shannon-Wiener Index (*H*), which was calculated from *H* = −∑*P*_*i*_ × ln*P*_*i*_ [12], where *P*_*i*_ is the importance probability of the bands in a gel lane, calculated as *P*_*i*_ = *n*_*i*_/*N*, where *n*_*i*_ is the intensity of a band and *N* is the sum of intensities of all bands.

### 2.5. Data Analysis

Three replicates were used in all parameter analyses. Data presented as mean values ± SD. One way analysis of variance (ANOVA) followed by *S*-*N*-*K*-test was performed to check for quantitative differences between samples; *P* < 0.005 was considered to be statistically significant. All statistical analyses were done using SPSS software.

The relative intensity of a specific band was transformed according to the sum of intensities of all bands in a pattern [13]. Redundancy analysis (RDA) for community ordination was conducted using CANOCO (version 4.5, Centre for Biometry, Wageningen, Netherlands) for Windows using relative band intensity data obtained from the Quantity One analysis [14]. Eight environmental parameters, including water temperature, pH, DO, ammonia, nitrate, nitrite, total nitrogen, and total soluble nitrogen, were selected to perform RDA-based variance inflation factor (VIF) analysis with 499 unrestricted permutations to statistically evaluate the significance of the first canonical axis and of all canonical axes together. Statistical significance was kept at *P* < 0.05 for all analyses.

## 3. Results

### 3.1. Water Properties

The corresponding environmental parameters (Table 2) of the eleven rivers represented their own properties of different pollution sources. The water from STP sites was characterized by relatively high concentrations of nitrate (4.79–12 mg L^−1^) and low concentrations of ammonia nitrogen (0.06–1.98 mg L^−1^) and organic matter, which had contrary properties comparing to those rivers receiving raw sewage from industrial, domestic, and agricultural sources. The XB and YA rivers had similar characteristics to those rivers receiving water from STP but lower dissolved oxygen and higher ammonia nitrogen.

### 3.2. Quantification of Denitrifying Genes (*nirK*,* nirS*,* nosZ*)

**Figure 2:**
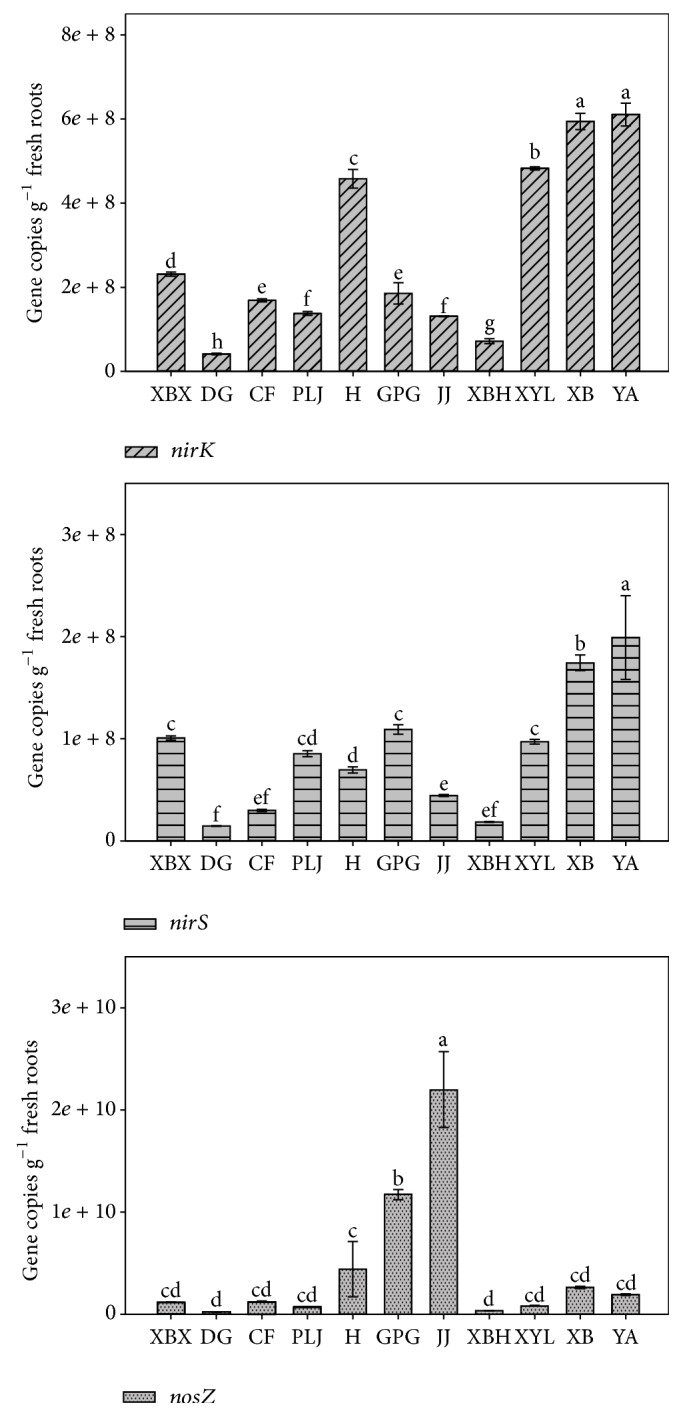
Abundance of* nirS, nirK,* and* nosZ* genes on the root of* E. crassipes*. Error bars indicate standard deviations (*n* = 3). The different letters indicate significant differences (*P* < 0.05).

The results showed that the abundance of* nirK, nirS, *and* nosZ* gene copies per gram fresh root ranged from 4.13 × 10^7^ to 6.11 × 10^8^, 1.45 × 10^8^ to 1.99 × 10^8^, and 2.20 × 10^8^ to 2.20 × 10^10^, respectively (Figure 2). The* nirK* and* nirS* abundance on the roots of* E. crassipes* in YA river and XB river were significantly higher than those in other rivers (*P* < 0.05). The highest abundance of* nosZ* was observed on the root sample in JJ river. The lowest abundance of* nirK* and* nosZ* type denitrifiers were determined on the root sample from DG river. The* nirK*,* nirS*, and* nosZ* copy abundance varied between sites indicated that different pollution source would influence the abundance of denitrifiers in rivers.

The highest abundance ratio (125.34) of* nosZ*/(*nirK* +* nirS*) occurred in JJ river, followed by GPG (39.75), while the lowest ratio was in XYL river (1.43), and the ratios in other rivers were similar, ranging from 3.26 to 8.38. However, the* nirK*/*nirS* ratio in all samples ranged from 1.70 to 6.60.

**Figure 3:**
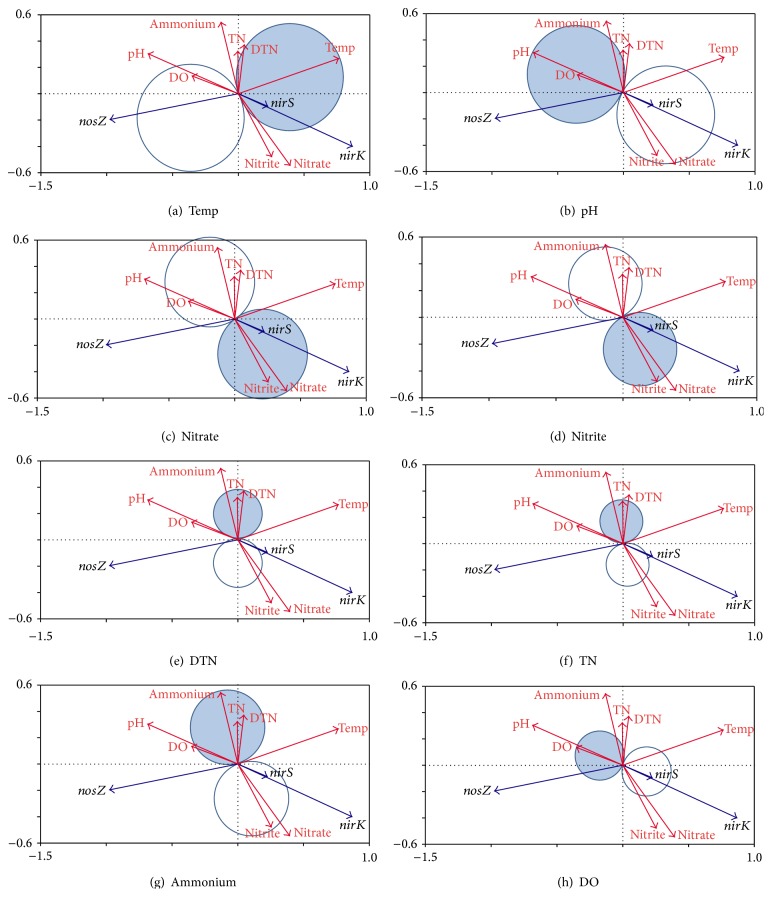
Redundancy analysis *t*-value biplots of environmental factors Temp, pH, nitrate, nitrite, DTN, TN, DO, and ammonium and abundance of* nirK*,* nirS,* and* nosZ* genes.

To explain the relationship between environmental factors and the abundance of* nirK*,* nirS*, and* nosZ*, the gene copy numbers of three denitrifiers and eight parameters were explored by redundancy analysis (Figure 3). The gray circle area implies a positive correlation and the white circle area implies a negative correlation. The larger the circle area, the greater the impact corresponding to the changes in environmental factors that would have influenced the denitrifiers. Denitrifier lines at the end in the gray circle had positive regression coefficients for that environmental variable with the corresponding *t*-value larger than 2.0. The results showed that the temperature, pH, and nitrate circle areas were larger than other environmental factors, which indicated that temperature, pH, and nitrate circle greatly affected the* nirS*,* nirK*, and* nosZ* abundance than other factors.

The abundance of* nirK* and* nirS* was positively correlated with water temperature, nitrate, and nitrite concentrations and was negatively correlated with the other factors (pH, DO, DTN, TN, and ammonium). The abundance of* nosZ* was negatively correlated with water temperature and was positively correlated with the pH and DO, while there were no significant correlations with other factors.

### 3.3. DGGE Fingerprints of* nirK*,* nirS*, and* nosZ* Genes

Only one of the three replicates of DGGE profiles was listed for each gene type to illustrate resolution. However, all the three replicates of the profiles were digitized and were used in statistics analysis.

### 3.4. Richness and Diversity of* nirK*,* nirS*, and* nosZ* on the Root of* E. crassipes*

The Shannon indices (*H*) calculated from DGGE gels ranged from 2.23 to 2.90 for* nirK*, 2.08 to 2.69 for* nirS*, and 2.11 to 2.73 for* nosZ*, which showed that high diversity of denitrifier (*nirK*,* nirS*, and* nosZ*) genes on the root of* E. crassipes* (Table 3). The significant differences among them were observed statistically (*P* < 0.05). With respect to the richness and diversity of denitrifier communities in all sites, similar trends emerged with low richness and diversity of* nirK* and* nirS* genes in XBX and DG rivers, which mainly received effluent from STPs. Next trends were in the H, JJ, and XBH rivers with relatively higher richness and diversity of* nirK* and* nirS* genes, which were less impacted by the effluent from STPs. The highest richness and diversity of* nirK* showed in the XB and YA rivers, which received wastewater after flowing through a wetland incubation on the water way. The richness and diversity of* nosZ*, which was mainly impacted by temperature, gave a similar trend for all rivers due to the fact that temperature did not vary too much in all sites.

### 3.5. Relationship between Environment Matrices and Denitrifier Diversity

To determine to what extent the eight environmental properties affected the three types of denitrifier community compositions,* nirK*,* nirS*, and* nosZ* DGGE fingerprints were evaluated by redundancy analysis (Table 4). The first axis explained 26.9% of the* nirK*-type denitrifier diversity, and the second axis explained 21.8% of the diversity. For* nirS*-type denitrifier, the first two canonical axes explained 29.5% and 11.5% of the variation, respectively. For* nosZ*-type denitrifier, 37.8% and 13.7% of the variation were explained by the first two canonical axes (Table 4).

Of the parameters, total N, DO, pH, and water temperature appeared to be the relatively important environmental factors for denitrifiers (Table 5). For* nirK*-type denitrifier, water temperature, DO, and total N explained 46% variations of microbial communities, leaving 54% of the variation unexplained. Variation partitioning analysis showed that water temperature, DO, and total N separately explained 19% (*P* = 0.020), 13% (*P* = 0.054), and 14% (*P* = 0.240) of the variation, respectively. For* nirS*-type denitrifier, water temperature (18%, *P* = 0.066), pH (10%, *P* = 0.304), and DO (8%, *P* = 0.038) explained 36% variations of microbial communities, leaving 64% of the variation unexplained. Compared to* nirS*, the total N rather than DO was relatively important for* nosZ*-type denitrifier (Table 5).

**Figure 4:**
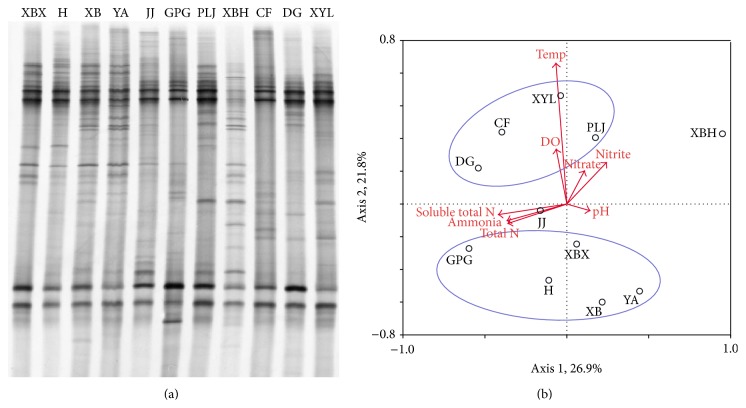
DGGE fingerprints and the redundancy analysis of DGGE band data of* nirK* gene. Arrows represent quantitative variables of environmental variables and small circles with letters represent the name of sample rivers; river names: XBX = Xinbaoxiang, DG = Daguang, CF = Chuangfang, PLJ = Panlongjiang, H = Haiheriver, GPG = Guangpugou, JJ = Jinjia, XBH = Xibahe, XYL = Xinyunling, XB = Xiaba, and YA = Yaoan.

**Figure 5:**
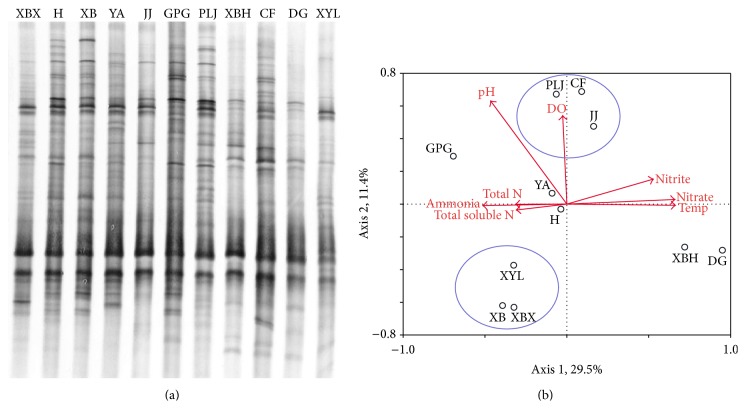
DGGE fingerprints and the redundancy analysis of DGGE band data of* nirS* gene. Arrows represent quantitative variables of environmental variables and small circles with letters represent the name of sample rivers; river names: XBX = Xinbaoxiang, DG = Daguang, CF = Chuangfang, PLJ = Panlongjiang, H = Haiheriver, GPG = Guangpugou, JJ = Jinjia, XBH = Xibahe, XYL = Xinyunling, XB = Xiaba, and YA = Yaoan.

**Figure 6:**
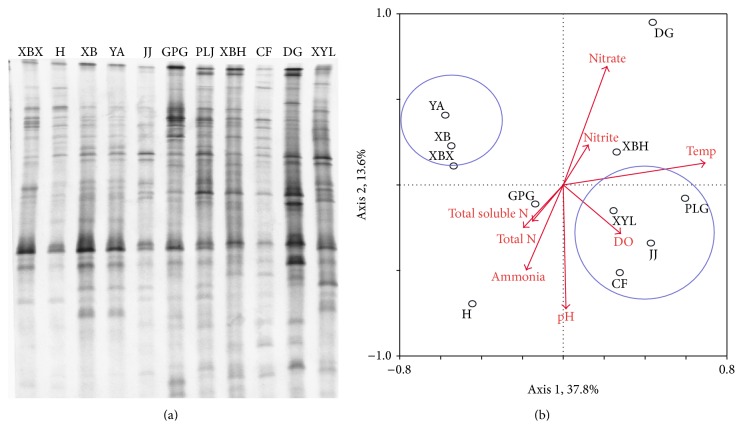
DGGE fingerprints and the redundancy analysis of DGGE band data of* nosZ* gene. Arrows represent quantitative variables of environmental variables and small circles with letters represent the name of sample rivers; river names: XBX = Xinbaoxiang, DG = Daguang, CF = Chuangfang, PLJ = Panlongjiang, H = Haiheriver, GPG = Guangpugou, JJ = Jinjia, XBH = Xibahe, XYL = Xinyunling, XB = Xiaba, and YA = Yaoan.

The relationships of microbial patterns to environmental variables were summarized in RDA ordination plots (Figures 4(b), 5(b), and 6(b)). The RDA charts (Figure 4(b)) of* nirK* gene showed four rivers (PLJ, XYL, DG, and CF) grouped into one type, while other four rivers (XBH, XBX, H, and YA) clustered together. Other rivers were located independently; they did not belong to either group. For* nirS* gene, PLJ, JJ, and CF rivers were similar and grouped into one type, while other three rivers (XBH, XYL, and XBX) clustered together. Other rivers were not similar and did not belong to either group (Figure 5(b)). According to the RDA chart (Figure 6(b)) of* nosZ* gene, DG, H, GPG, and XBH rivers were located independently and did not cluster with any group. However, PLJ, XYL, JJ, and CF rivers clustered into one group, while other three rivers (XB, XBX, and YA) clustered together.

## 4. Discussion

The activity of denitrifying microorganisms leads to significant net removal of dissolved nitrogen from the water, resulting in considerable improvement of water quality in aquatic ecosystem [15]. Denitrifiers play an important role in buffering of the excessive load of nitrogen from upstream to downstream [16]. In aquatic ecosystems, mats of macrophytes are important sites for microbial mediated biogeochemical processes, as accrual of biomass and increases in mat density reduce the degree of external factors to influence internal processes [17]. The suspended root system of* E. crassipes* could provide a large surface area, approximately 2.5 to 8.0 m^2 ^kg^−1^ on a dry weight basis, for microbial attachment [5, 18]. Releasing of oxygen and dissolved organic carbon from roots of* E. crassipes* would support an appropriate microenvironment for nitrification and/or denitrification [19, 20].

Process of denitrification is driven by the denitrifying microorganisms under the influence of environmental conditions. Water properties in different rivers in the present study were shown altering the abundance and diversity of denitrifiers on* E. crassipes* roots.

### 4.1. Differential Characteristics of Different Rivers Impact the Abundance of Denitrifier on the Roots

The abundance of* nirK*,* nirS*, and* nosZ* denitrifiers on the root of macrophytes varied with the variation of environmental parameters in different rivers, which seemed depending on the nitrogen concentrations, water temperature [21], water turbulence, and pretreatment of wastewater using wetland.

The abundance of* nirK*,* nirS*, and* nosZ* denitrifiers on root samples from XBX, DG, CF, and PLJ rivers was relatively stable and low. These rivers were larger than other rivers around Dianchi Lake [22], which were important sites receiving effluent from the STPs. The fast-flowing water and irregular discharge of effluent of these rivers [22] may prevent development of the stable environment properties from microbial attachment and propagate. Contrarily, the abundance of denitrifiers genes on roots samples in XB and YA rivers (Xiaba and Yaoan rivers) was higher than that in other rivers. The XB river received both the wastewater from industrial and residential areas and the tidal water from Dianchi Lake, when water level increased in rainy seasons (May to October) [23]. The water merged at Wujia wetland, and then part of it was pumped into YA river after 45 days of retention in Wujia wetland. This implies that a combination of wetland and growth of water hyacinth may further promote denitrification processes in eutrophic water.

The abundance of* nirK* gene was always greater than that of* nirS* gene on the roots of* E. crassipes*, suggesting that the fresh water of Dianchi lake was more suitable for the growth of* nirK*-type denitrifying bacteria. This was different from the previous reports [24]. The* nirS* gene of cytochrome* cd1* type has also been found more often in anoxic locations, where DO levels were consistently low. In contrast,* nirK* genes of copper containing type have been found where diurnal DO swings are greater [24, 25]. This finding was of coincidence with that* E. crassipes* releases oxygen from roots, which facilitates the creation of aerobic microsites on the roots [26]. Even though the* nirK* and* nirS* are functionally equivalent, denitrifying bacteria harboring either nitrite reductase seems to be likely not under the same community assembly rules [27]. Philippot et al. [28] suggested that the existence of the two types of nitrite reductase (*nir*-gene) was due to differential niche preferences. This speculation was consistent with previously identified habitat preferences of* nirS*-gene and* nirK*-gene bearing organisms [29]. Moreover,* nirK* and* nirS* sequences may come from different sources. Jones and Hallin [27] found that most* nirK* sequences were derived from soil but that most* nirS* sequences were prominently derived from marine and estuarine environment. Bacteria suspended in water and attached to the root of* E. crassipes* may originate from many different sources. Autochthonous bacterioplankton populations that developed in the water column were likely to be mixed with allochthonous populations from forest soils, urbanized land, farm fields, and wetlands as well as hyporheic sediments in the rivers. This mixed origination, impacted by varied environmental parameters, seemed to be the main cause of the discrepancy of denitrifiers found in the eleven rivers. This, however, did not necessarily indicate that* nirK*-type denitrifiers contributed more or less in denitrification than* nirS*-type ones; rather it may only imply that the root of* E. crassipes* could provide a broad support for different kinds of microorganisms.

### 4.2. Relationship between Environmental Factors and Community Compositions of Denitrifying Genes on* E. crassipes* Root at Different Rivers

Dianchi Lake together with surrounding rivers comprised a plateau water catchment to provide ecological services and fresh water supply for more than seven million people in the area. Its geochemical characteristics have made the water pH relatively high (7.56–8.03) and its geophysical characteristics have made the water temperature moderate with winter months (December to March next year) around 12°C. Its heavy load of organic matters have made the DO level relatively low (0.20–3.80 mg L^−1^) in the 11 rivers investigated. These environmental properties dominated the community assembly processes of the genetic makeup of the denitrifiers in the rivers. Nevertheless, specific environmental conditions in different rivers favored the variation in richness and diversity of different denitrifying genes.

The DGGE profiles for denitrification genes encoding nitrite and nitrous oxide reductase (*nirK*,* nirS*, and* nosZ*) on the root of* E. crassipes* growing in 11 rivers around Dianchi Lake supported our hypothesis of profound differences in community composition, although a complex picture of denitrifier community similarity emerged depending on which functional denitrification gene was evaluated. The correlations of denitrifying microbial community compositions with abiotic environmental factors, using redundancy analysis (RDA), confirmed that water temperature (Temp), dissolved oxygen (DO), and pH appeared to be the most important factors to alter the denitrifier community structures significantly by serving as essential conditions for the growth of microorganisms on the roots of* E. crassipes* (Table 5).

The results of this study indicated that the development of denitrifier communities on roots corresponded to different origins of rivers. The physiochemical characteristics of water from the river inlet varied with water origin and pollution sources [22, 23, 30], resulting in the variation in DO, pH, pollutant species and concentrations, and organic carbons in rivers. These environmental factors, including DO, carbon content, water temperature, and pH, influenced denitrification rates in rivers [31] and as a consequence they might also affect the denitrifier community composition [32, 33]. Braker et al. [34] found that the change of temperature resulted in gradually changed denitrification activity but also in abundance mutative of nitrate reducers and in different denitrifier community compositions. There are some indications that temperature and pH may directly or indirectly influence the abundance and communities composition of denitrifiers [35, 36]. The excess O_2_ resulted in reduced denitrifying bacterial growth and a smaller bacterial density versus nitrate reducing bacteria ration [37], which indicated that the development of the denitrifying bacteria was influenced by the DO concentration. Many investigators had found that the pH, temperature, and DO generally affect diversity and richness of denitrifier community [32, 38], and microbial community assembly was more dependent on local-scale environmental factors [39].

On the other hand, different microorganisms may have their physiological constraints for growth and reproduction within narrow pH ranges, specific DO, and nutrient availability, which affect the community structures directly [40, 41]. Activities of microorganisms could change the environmental properties that differed in the concentrations of enzymes and nutrients or DO, the form and amount of dissolved carbon present, and pH [42] hence to affect denitrifier community structure. Previous studies have found that growth of* E. crassipes* could regulate water at neutralize pH significantly [43], as a result of increase in the rate of denitrification in aquatic ecosystems [44].

## 5. Conclusions

The variation in abundance of denitrifier communities on* E. crassipes* roots, grown in rivers flowing into Dianchi Lake, corresponded to different water properties of rivers. The ratio of* nirK*/*nirS* gene copies abundance was always greater than 1, indicating that the surface of* E. crassipes* roots was more suitable for the growth of* nirK*-type denitrifying bacteria. The temperature of water, nitrate concentration, and pH greatly affected the* nirS*,* nirK*, and* nosZ* abundance than other factors. Meanwhile, the temperature of water, DO, and pH appeared to be the most important factors to alter the community structures of denitrifiers on the roots of* E. crassipes*. As process of denitrification is driven by denitrifies under the influence of environmental conditions, a variation of denitrification capability in different rivers would be expected.

## Figures and Tables

**Table 1:** Primers used for the qPCR and DGGE.

Gene		Primers	Thermal profile
*nosZ *	for qPCR for DGGE	*nosZ*-F [45]	CGYTGTTCMTCGACAGCCAG
		*nosZ*1622R [45]	CGSACCTTSTTGCCSTYGCG
		*nosZ*1622R-GC [46]	GGCGGCGCGCCGCCCGCCCCGCCCCCGTCGCCCCGSACCTTSTTGCCSTYGCG

*nirS *	for qPCR for DGGE	*nirS*-Cd3Af [47]	GTSAACGTSAAGGARACSGG
		*nirS*-R3cd [47]	GASTTCGGRTGSGTCTTGA
		*nirS*-R3cd-GC [48]	GGCGGCGCGCCGCCCGCCCCGCCCCCGTCGCCCGASTTCGGRTGSGTCTTGA

*nirK *	for qPCR for DGGE	*nirK* F1aCu [49]	ATCATGGTSCTGCCGCG
		*nirK* R3Cu [49]	GCCTCGATCAGRTTGTGGTT
		*nirK* R3Cu-GC [49]	GGCGGCGCGCCGCCCGCCCCGCCCCCGTCGCCC- GCCTCGATCAGRTTGTGGTT

**Table 2:** Water properties of the rivers at Dianchi Lake (mean ± SD).

Rivers	NO_3_^−^ (mg L^−1^)	NO_2_^−^ (mg L^−1^)	NH_4_^+^ (mg L^−1^)	TN (mg L^−1^)	TSN (mg L^−1^)	DO (mg L^−1^)	pH	*T* (°C)
XBX	4.79 ± 0.39	0.25 ± 0.02	0.69 ± 0.07	7.44 ± 0.19	6.54 ± 0.32	2.15 ± 0.15	7.83 ± 0.06	19.60 ± 0.00
DG	12.00 ± 0.67	0.46 ± 0.00	0.10 ± 0.02	12.91 ± 0.62	12.26 ± 0.42	2.70 ± 0.13	7.56 ± 0.04	22.15 ± 0.05
CF	5.09 ± 0.03	0.62 ± 0.02	1.98 ± 0.02	8.63 ± 0.10	7.57 ± 0.06	3.80 ± 0.10	7.90 ± 0.06	21.75 ± 0.05
PLJ	5.86 ± 0.01	0.10 ± 0.00	0.06 ± 0.02	8.08 ± 0.19	6.45 ± 0.08	2.80 ± 0.00	8.03 ± 0.00	19.70 ± 0.00

H	0.16 ± 0.02	0.09 ± 0.00	18.48 ± 2.30	23.69 ± 0.79	19.84 ± 1.91	0.20 ± 0.10	7.90 ± 0.01	18.80 ± 0.00
GPG	0.08 ± 0.02	0.04 ± 0.01	19.54 ± 0.19	23.66 ± 0.16	21.27 ± 0.07	0.20 ± 0.10	7.83 ± 0.00	17.90 ± 0.00
JJ	0.43 ± 0.02	0.15 ± 0.00	5.12 ± 0.50	8.14 ± 0.54	6.86 ± 0.37	1.30 ± 0.10	7.90 ± 0.01	19.50 ± 0.00
XBH	5.92 ± 0.25	0.70 ± 0.00	0.78 ± 0.20	8.60 ± 0.19	6.96 ± 0.58	0.60 ± 0.00	7.77 ± 0.00	21.60 ± 0.00
XYL	0.08 ± 0.02	0.05 ± 0.00	18.72 ± 3.31	21.40 ± 2.78	19.95 ± 3.55	0.45 ± 0.05	7.78 ± 0.02	21.55 ± 0.05

XB	0.56 ± 0.14	0.17 ± 0.04	3.03 ± 0.52	6.35 ± 1.21	5.60 ± 0.64	0.55 ± 0.25	7.78 ± 0.06	17.95 ± 0.05
YA	8.99 ± 0.00	0.62 ± 0.01	2.49 ± 0.27	15.08 ± 0.50	11.97 ± 0.56	1.05 ± 0.35	7.85 ± 0.07	17.60 ± 0.10

**Table 3:** Shannon (*H*) and richness (*S*) values of *nirK*, *nirS*, and *nosZ* genes.

Rivers	*nirK *		*nirS *		*nosZ *	
	*S*	*H*	*S*	*H*	*S*	*H*
XBX	11.00 ± 1.00	2.25 ± 0.20	11.67 ± 0.58	2.18 ± 0.06	10.67 ± 0.58	2.11 ± 0.10
DG	13.67 ± 1.15	2.23 ± 0.21	11.67 ± 1.53	2.15 ± 0.07	15.67 ± 0.58	2.56 ± 0.20
CF	16.67 ± 0.58	2.48 ± 0.17	15.33 ± 0.58	2.57 ± 0.30	17.33 ± 0.58	2.65 ± 0.25
PLJ	16.67 ± 1.15	2.54 ± 0.41	18.67 ± 1.15	2.65 ± 0.21	18.00 ± 1.00	2.73 ± 0.26
H	17.00 ± 0.00	2.53 ± 0.45	17.67 ± 1.53	2.69 ± 0.31	11.00 ± 0.00	2.18 ± 0.16
GPG	14.33 ± 1.53	2.36 ± 0.37	17.00 ± 0.00	2.66 ± 0.34	16.33 ± 0.58	2.44 ± 0.34
JJ	18.66 ± 058	2.59 ± 0.04	10.33 ± 1.53	2.08 ± 0.35	12.33 ± 0.58	2.25 ± 0.21
XBH	17.67 ± 0.58	2.71 ± 0.19	13.33 ± 0.58	2.26 ± 0.19	14.67 ± 0.58	2.45 ± 0.32
XYL	14.33 ± 1.15	2.38 ± 0.16	13.00 ± 0.00	2.56 ± 0.22	16.33 ± 0.58	2.64 ± 0.24
XB	21.00 ± 1.00	2.61 ± 0.20	17.00 ± 0.00	2.64 ± 0.39	14.67 ± 1.15	2.35 ± 0.31
YA	22.33 ± 0.58	2.90 ± 0.56	15.33 ± 0.00	2.54 ± 0.11	13.00 ± 0.00	2.29 ± 0.15

**Table 4:** Redundancy analysis results of *nirK*, *nirS*, and *nosZ* DGGE profiles.

Axis	Eigenvalue	Denitrifier-environment correlation	Cumulative% variation of denitrifier	Cumulative% variation of denitrifier-environment	Sum of all canonical eigenvalues
*nirK* RDA					
Axis 1	0.269	0.987	26.9	31.0	0.867
Axis 2	0.218	0.954	48.7	56.2	
Axis 3	0.141	0.944	62.8	72.5	
Axis 4	0.075	0.990	70.3	81.1	

*nirS* RDA					
Axis 1	0.295	0.965	29.5	42.6	0.693
Axis 2	0.114	0.895	41.0	59.1	
Axis 3	0.099	0.780	50.8	73.3	
Axis 4	0.072	0.861	58.0	83.8	

*nosZ* RDA					
Axis 1	0.378	0.996	37.8	48.1	0.786
Axis 2	0.136	0.973	51.5	65.5	
Axis 3	0.097	0.682	61.2	77.9	
Axis 4	0.071	0.971	68.4	87.0	

**Table 5:** Eigenvalues, *F* values, and *P* values obtained from the partial RDAs testing the influence of the significant water properties on the denitrifying bacterial community composition.

Samples	Environmental variables	Eigenvalue	% variation explains solely	*F* value	*P* value
*nirK *	Temp	0.19	19	2.08	0.020
	DO	0.13	13	1.46	0.054
	Total *N*	0.14	14	1.54	0.240
	All the above together	0.92	92		

*nirS *	Temp	0.18	18	1.93	0.066
	pH	0.10	10	1.19	0.304
	DO	0.08	8	0.96	0.038
	All the above together	0.69	69		

*nosZ *	Temp	0.22	22	2.50	0.032
	pH	0.11	11	1.41	0.082
	Total *N*	0.13	13	1.39	0.260
	All the above together	0.79	79		

Partial RDAs based on Monte Carlo permutation (*n* = 499) kept only the significant water properties in the models. For each partial model, the other significant water properties were used as covariables. *F* and *P* values were estimated using Monte Carlo permutations. Sum of all eigenvalues for both partial RDAs was 1.000.
